# Myotonic disorders: A review article

**Published:** 2016-01-05

**Authors:** Chris Hahn, Mohammad Kian Salajegheh

**Affiliations:** ^1^ Department of Neurology, Brigham and Women’s Hospital, Harvard Medical School, Boston, USA

**Keywords:** Myotonia, Myotonic Dystrophy, Myotonia Congenita, Paralysis Periodica Paramyotonia, Hyperkalemic Periodic Paralysis

## Abstract

The myotonic disorders are a heterogeneous group of genetically determined diseases that are unified by the presence of myotonia, which is defined as failure of muscle relaxation after activation. The presentation of these disorders can range from asymptomatic electrical myotonia, as seen in some forms of myotonia congenita (MC), to severe disability with muscle weakness, cardiac conduction defects, and other systemic features as in myotonic dystrophy type I (DM1). In this review, we describe the clinical features and pathophysiology of the different myotonic disorders, their laboratory and electrophysiologic findings and briefly review the currently available treatments.

## Introduction

The myotonic disorders are a group of rare, genetically heterogeneous syndromes presenting with clinical and/or electrical myotonia. Clinical myotonia is characterized by the failure of muscle relaxation after activation.^1^ Electrical myotonia is the spontaneous discharge of muscle fibers that waxes and wanes in both amplitude and frequency on electromyography (EMG). Myotonia is thought to be due to increased excitability of muscle fibers, leading to discharge of repetitive action potentials in response to stimulation.^2^ Electrical myotonia can also be seen with certain drugs (cholesterol lowering agents, cyclosporine, and colchicine, among others), in inflammatory myopathies, Pompe disease, hypothyroidism, myotubular myopathy, and chronic denervation (usually as brief runs).^1^

Clinical myotonia manifests with painless muscle stiffness, although some forms can be associated with pain.^2^^,^^3^ The typical location of stiffness varies depending on the underlying disorder but commonly seen in the eyelids, mouth, hands, and proximal legs.^3^^-^^6^ Common triggers include cold, stress and exercise, and symptoms can worsen during pregnancy and menstruation.^3^ Most demonstrate a “warm-up” phenomenon, where myotonia improves with repeated action.^3^^,^^5^^,^^6^ In contrast, paradoxical myotonia or paramyotonia worsens with repeated use. Some forms of myotonia are also associated with diffuse muscle hypertrophy.^7^

Myotonia can be brought out by asking the patient to repeatedly grip and relax their hand or open and close their eyes. Alternatively, direct percussion of a muscle can achieve the same effect; including tapping the thenar eminence, forearm extensors, or even tongue.^8^

Typically, myotonias are classified as either dystrophic or non-dystrophic (table 1). The former are characterized by fixed muscle weakness, systemic features, and dystrophic changes on muscle biopsy. Fixed weakness and dystrophic changes are less common, but can be seen in the non-dystrophic myotonias (NDM), and myopathic changes may be noted on muscle biopsy.^9^ Recent evidence suggests structural muscles changes on magnetic resonance imaging and ultrasound imaging of some patients.^7^^,^^10^

**Myotonic dystrophy**

The myotonic dystrophies are inherited in an autosomal dominant fashion and classified into type I (DM1) and type II (DM2), also known as proximal myotonic myopathy (PROMM).

**Table 1:** Clinical features of familial myotonic disorders

**Conditions**	**Inheritance**	**Gene**	**Myotonia**	**Episodic weakness**	**Fixed weakness**	**Major trigger**	**Other features**
DM							
DM type 1	AD	DMPK	M	Absent	Distal limbs, face	None	Frontal balding, temporal wasting, cataracts, systemic disease
Myotonic dystrophy type 2 (PROMM)	AD	CNBP (ZNF9)	M	Absent*	Proximal limbs	None	Disabling and atypical pain, cataracts, milder systemic disease
NDM							
MC							
AD (Thomsen)	AD	CLCN1	M	Absent	Rare	Rest	Generalized muscle hypertrophy
AR (Becker)	AR	CLCN1	M	Absent*	Proximal LE	Rest	Muscle hypertrophy in LE
PMC	AD	SCN4A	P	Present in some	Proximal LE	Cold, exercise	Most sensitive to cold
Potassium-sensitive periodic paralysis	AD	SCN4A	P, M or absent	Present	Proximal LE	K+, rest after exercise	Potassium levels may be high***
PAM							
Myotonia fluctuans	AD	SCN4A	M**	Absent	Absent	K+, exercise	Have good days and bad days
Myotonia permanens	AD	SCN4A	M**	Absent	Absent	K+, exercise	Continuous muscle stiffness
Acetazolamide-responsive myotonia	AD	SCN4A	M**	Absent	Absent	K+, exercise	Respond to therapy with acetazolamide

AD: Autosomal dominant; AR: Autosomal recessive; K+: Potassium, LE: Lower extremities; M: Myotonia; P: Paramyotonia; PROMM: Proximal myotonic myopathy; DM: Dystrophic myotonias

* PROMM patients may initially have intermittent or transient weakness; recessive MC patients may have transient weakness after severe bouts of stiffness,

** May have eyelid paramyotonia,

*** Potassium levels may be normal during attack normokalemic periodic paralysis (normoKPP).

Both are caused by expansion of DNA tandem repeats, resulting in a toxic gain of function of the resulting mutant RNA and sequestration of RNA-binding proteins.^11^ DM1 results from expansion of CTG repeats in the DM protein kinase (DMPK) gene^12^^,^^13^ whereas DM2 is caused by expansion of CCTG repeats in the ZNF9 gene.^14^^,^^15^

**DM1**

DM1 has an estimated prevalence of 3-15 per 100000^16^^,^^17^ with higher prevalence reported in Sweden, the Basque area of Spain, and the Saguenay region of Quebec, Canada, were it is up to 20 times higher.^16^^,^^18^ To our knowledge, disease prevalence has not been reported in Iran, but the incidence of excess DMPK CTG repeats in healthy Iranian controls is reported to be similar to Western Europe and Japan.^19^

DM1 can present at any age and often occurs earlier in successive generations, showing marked anticipation.^20^ Larger repeat expansions are associated with earlier onset and more severe forms of the disease in most cases.^20^^-^^25^ The most severe form, congenital DM, presents at birth with generalized hypotonia, severe weakness, facial diplegia (tent shaped mouth), intellectual disability, hypoventilation, gastrointestinal (GI) dysmotility, and early death.^17^^,^^23^ Interestingly, myotonia is usually absent in infancy and muscle strength can improve with time if the infant survives.

Classically, DM1 presents in the second to fourth decade with prominent facial weakness, including ptosis, neck extension/flexion weakness, and distal weakness with a predilection for finger flexors/extensors and foot dorsiflexors.^20^ Muscle atrophy occurs in line with the progression of weakness and myotonia is common (particularly in the hands).^17^^,^^22^^,^^25^ Temporal muscle atrophy, ptosis and frontal balding result in a characteristic myotonic facies, and dysphagia and dysarthria are often prominent. Weakness tends to progress slowly over time with more than 95.0% of patients remaining ambulatory after a mean disease duration of 16-19 years.^20^^,^^26^

Systemic features include cardiac disease, especially conduction defects and possibly cardiomyopathy.^27^ Cataracts are seen early (before 50 years in most) with bilateral iridescent (Christmas tree) or posterior cortical lens opacities being highly specific for DM1.^28^ GI symptoms have been attributed to smooth muscle dysfunction and include reflux, abdominal pain, bloating, constipation, and diarrhea.^29^ Endocrinopathies, in the form of insulin resistance and hypogonadism may also occur.^30^ Excessive daytime sleepiness, independent of obstructive sleep apnea,^31^ is common. Patients may have mild cognitive impairment and often have an avoidant personality with apathy toward their disease.^32^^,^^33^ More recently, associations with mild peripheral neuropathy^34^ and increased risk of cancer have been suggested.^35^ Mild forms, limited to myotonia, frontal balding and early cataracts with normal strength and lifespan also occur, usually in patients with lower number of repeats.^20^^,^^22^

**DM2**

The prevalence of DM2 or PROMM is unclear but reported as even higher than DM1 in some populations in Finland and the Czech Republic.^36^^,^^37^ Unlike DM1, there is no clear relationship between the number of repeats and disease severity^36^ and congenital forms have not been reported. Most patients present in middle age with a mild phenotype of proximal weakness, myalgias, and early cataracts. Weakness characteristically involves neck flexors, elbow extensors, thumb/deep finger flexors, hip flexors and hip extensors. Facial weakness is less common.^38^ Marked muscle atrophy is uncommon, and calf hypertrophy can be seen in some.^37^ Muscle pain is present in 56-76%^38^^,^^39^ of patients and occasionally leads to a misdiagnosis of fibromyalgia.^40^^,^^41^ Clinical myotonia is often absent, and patients can occasionally present with asymptomatic hyperCKemia.^37^^,^^38^^,^^42^

Systemic features are similar to DM1 but milder.^35^^,^^38^^,^^43^^,^^44^ Cognitive function is mostly normal although mild changes have been reported.^45^^-^^47^ Dysphagia is common but typically mild.^48^^,^^49^ In addition, there are reports of higher incidence of autoimmune disease compared to controls and DM1.^50^

**Diagnosis and treatment of DMs**

The evaluation of patients suspected to have myotonic dystrophy should include a thorough family history and physical examination, including testing of strength and myotonia. Definitive diagnosis is through genetic testing, commercially available for both DM1 and DM2. CK levels can be normal to moderately elevated,^38^^,^^39^ and other laboratory features include mild hypogammaglobulinemia and non-specific liver enzyme abnormalities.^37^ Nerve conduction studies are usually normal but can show mild length dependent axonal polyneuropathy. EMG shows myotonic discharges in all patients with DM1 and 90-100% of patients with DM2.^38^^,^^51^^,^^52^ Myopathic units, fibrillation potentials, and positive sharp waves can also be seen but are sometimes obscured by prominent myotonic discharges. Muscle biopsy in DM1 shows atrophy of type I fibers, increased internalized nuclei, ring fibers, sarcoplasmic masses, small angulated fibers, and atrophic fibers with pyknotic clumps.^16^^,^^17^ DM2 may show similar features but milder and with atrophy of type II fibers, in contrast.

Treatment of the myotonic dystrophies is symptomatic and should include screening for cardiac arrhythmias, glucose intolerance, obstructive sleep apnea, and cataracts. Limited evidence exists for drug treatment of myotonia with antiepileptic and antiarrhythmia agents.^53^ A recent randomized controlled trial demonstrated efficacy of mexiletine in patients with DM1;^54^ however, concerns have been raised regarding its long-term safety in DM1 patients due to the potential of cardiac arrhythmias.^55^ We use it with caution in patients with DM1 and in consultation with cardiology. It may be started at 150 mg twice daily and slowly increased to a maximum of 300 mg 3 times daily. An electrocardiogram should be performed at baseline and periodically.

Excessive daytime somnolence is sometimes treated with stimulants, although evidence for efficacy is mixed.^56^^-^^59^ GI complaints can occasionally be due to small intestine bacterial overgrowth and may respond to antibiotic therapy,^60^ and prokinetic agents have been used to treat gastroparesis.^61^

**Non-Dystrophic Myotonias** **‎** **(NDM)**

These are a group of rare disorders caused by mutations in genes coding for sodium (SCN4A) or chloride (CLCN1) channels. Their prevalence is estimated at ~1/100000 with higher rates (7-9/100000) in Northern Finland and Norway.^62^^-^^65^ CLCN1 mutations are the most common form of NDM (0.52/100000) followed by paramyotonia congenita (PMC) (0.17/100000).^65^

The muscle chloride channel was previously thought to be important in stabilizing the resting membrane potential, with mutations leading to a lower threshold for muscle membrane firing.^66^ More recent studies bring this into question.^67^ Sodium channel mutations lead to poor inactivation of the channel, which results in repetitive discharges, with mild depolarization, or weakness, with severe depolarization.^68^^,^^69^

**Myotonia congenita (MC)**

MC is a chloride channelopathy, typically divided into dominant (Thomsen disease) and recessive (Becker disease) forms. Over 150 mutations in the CLCN1 gene have been reported,^70^ with the recessive form of the disease classically presenting earlier in life with a more severe phenotype, although mild or late presentations have been reported.^71^^,^^72^

Thomsen disease was initially described by Thomsen himself in 1876, through a detailed description of his own disability and that of his family members.^73^ It typically presents in the first decade with stiffness in legs more than arms, hands, and face.^3^^,^^6^^,^^72^ Penetrance is incomplete, and disease severity can vary widely in family members.^74^ Classically, stiffness is worse after rest, and patients often complain of leg stiffness that improves with walking. Myotonia can also affect muscles of mastication and swallowing. Some patients describe pain associated with their stiffness.^3^^,^^6^ Warm-up phenomenon is usually present, and the stiffness can worsen with cold exposure, pregnancy, menses, hypothyroidism, and stress.^3^^,^^6^^,^^17^^,^^75^^,^^76^ Examination demonstrates generalized muscular hypertrophy, action myotonia more pronounced in the hands than eyelids,^3^^,^^6^ and percussion myotonia. Thomsen disease is not associated with systemic features, and patients have normal lifespans.

Becker disease usually presents between the ages of 4 and 12 years, although onset in adulthood is also seen.^72^^,^^73^ Symptoms are similar to Thomsen disease, but myotonia tends to be more severe in the lower limbs and proximal muscles, and men tend to be more severely affected than women.^72^ Transient weakness, lasting seconds to minutes, is sometimes seen after sustained bouts of myotonia and patients often have difficulty moving if startled suddenly.^6^^,^^73^ Patients may develop mild fixed distal weakness and a “dystrophic” variant with severe atrophy and weakness, contractures and myopathic changes on muscle biopsy.^77^ Similar to Thomsen disease, no systemic features are seen, and lifespan is normal.

Laboratory investigations are usually normal in both dominant and recessive MC although mild elevations in CK can be seen.^73^ EMG shows widespread myotonic discharges and myopathic motor units can be seen in weak muscles if not obscured by myotonia. Given the commercial availability of genetic testing, muscle biopsy is rarely done and shows non-specific changes or mildly increased variability in fiber size and increased number of internalized nuclei.^73^

**Paramyotonia Congenita** **‎(PMC)**

PMC is an autosomal dominant condition caused by mutations in the SNC4A gene. It is highly penetrant and typically presents in the first decade with stiffness that is most pronounced in the face and hands. In contrast to other forms of myotonia, the patients have paramyotonia.^3^^,^^6^ It commonly worsens with cold and patients can develop severe weakness with the prolonged exposure that can take hours to improve despite rewarming.^17^^,^^78^^,^^79^ Patients may complain of hand stiffness while shoveling snow or in the frozen food section of the supermarket. Parents will occasionally report that affected infants are unable to open their eyes after a crying spell, presumably due to the eyelids being “exercised” while crying.

Myotonia can worsen with pregnancy, menstruation, ingestion of potassium-rich food, and anesthetics.^3^^,^^78^ Muscle hypertrophy is less common than in MC, and some patients develop progressive weakness with time.^78^ This disorder is allelic with potassium-sensitive periodic paralysis, and some patients demonstrate features of both disorders with episodes of generalized weakness.^9^^,^^79^^,^^80^ Lifespan is unaffected and systemic involvement is not a feature of PMC.^81^

Examination of affected patients shows eyelid and grip paramyotonia in most patients,^78^ but percussion myotonia is not prominent. Immersing a limb in cold water can worsen myotonia and result in weakness. Laboratory investigations show mild elevations in CK, and potassium levels can be high or normal during episodes of weakness.^79^^,^^82^ EMG shows diffuse myotonic discharges, which can worsen with cooling of the affected limb, as well as eventual electrically silent contractures with progressive cooling.^83^ Genetic testing is commercially available, and muscle biopsy demonstrates non-specific myopathic changes with occasional vacuoles.^9^^,^^82^

**Potassium aggravated myotonia (PAM)**

These are a group of autosomal dominant NDMs characterized by sensitivity to potassium ingestion without episodic weakness. They are caused by mutations in SCN4A and include myotonia fluctuans, myotonia permanens, and acetazolimide responsive myotonia. CK levels can be normal or mildly elevated, and EMG shows diffuse myotonic discharges and fibrillation potentials. Commercial genetic testing is available, and changes on muscle biopsy are not well-described.^84^^-^^87^

Myotonia fluctuans typically presents in the first to the second decade of life, with myotonia that fluctuates from day-to-day. Patients can be asymptomatic 1 day and have severe myotonia affecting the limbs, extraocular muscles, muscles of mastication, and swallowing on other days. Myotonia is accompanied by “warm-up” phenomenon and increases with potassium ingestion and with a short delay (usually minutes) after exercise. Patients do not develop fixed weakness, and there is no increase in myotonia with exposure to cold.^84^^,^^85^^,^^88^ Eyelid paramyotonia is frequently seen on examination while grip myotonia is less common.

Myotonia permanens is characterized by constant, generalized myotonia that can affect respiration and even lead to hypoxia and respiratory acidosis.^68^^,^^86^ It has been reported with neonatal episodic laryngospasm,^89^ and to worsen with potassium ingestion, fever, pregnancy and after exercise,^86^^,^^87^ without associated weakness or exacerbation with cooling.

Acetazolimide responsive myotonia presents as painful muscle stiffness in childhood that can involve proximal limbs, muscles of mastication, and extraocular muscles. It worsens with potassium and fasting but may improve with carbohydrate ingestion. Examination shows variable muscle hypertrophy, easily elicitable action and percussion myotonia and paradoxical myotonia in the eyelids.^90^^,^^91^

**Potassium-sensitive periodic paralysis**

This disorder, also called hyperkalemic periodic paralysis (HyperPP), is associated with autosomal dominant mutations in SCN4A. It can present as a pure periodic paralysis syndrome or as periodic paralysis with clinical/electrical myotonia or paramyotonia.^17^ Myotonia is usually mild, often involving the eyelids, hands, and tongue. The attacks of weakness can occur at any time and are commonly triggered by rest the following exercise, fasting, ingestion of food high in potassium or stress.^92^ Some patients may develop progressive myopathy.^93^

**Other sodium channel myotonias**

Severe neonatal episodic laryngospasm is a recently described entity consisting of recurrent laryngospasm which results in apnea and apparent life-threatening events in neonates. This has been associated with multiple mutations in SCN4A.^89^^,^^94^^,^^95^ It remains unclear if this is a de novo disorder or represents a neonatal presentation of NDM.

**Diagnosis and treatment of NDM**

Clinical examination and electrodiagnostic studies are helpful in narrowing down the differential before ordering commercially available genetic tests. Pronounced sensitivity to cold is most suggestive of PMC, although mild cold intolerance is present in other forms. Myotonia tends to be more prominent in the legs in MC, leading to difficulty standing up quickly, while more prominent in the arms and face in PAM. Eye closure myotonia, as well as grip and eye closure paramyotonia, are more common with SCN4A mutations and warm-up phenomenon with MC.^3^^,^^6^ Patients with recessive MC,^96^ PMC, and HyperPP are more likely to report episodes of transient paresis.

The degree of myotonic discharges does not seem to differ greatly between MC and sternocleidomastoid on EMG, but may be less in DM2.^3^^,^^97^

The use of the short exercise test, in particular with cooling, has proven useful in differentiating various forms of myotonia.^98^^,^^99^ The long exercise test can also be helpful, but typically used for diagnosing periodic paralysis.^97^

Treatment of NDM includes the avoidance of triggers and, if necessary, symptomatic treatment of myotonia. PMC patients should be counseled to avoid cold exposure, and HyperPP, PMC or PAM patients to avoid foods rich in potassium (banana, papaya, mango, beans, and dried fruits). Mexiletine use for treatment of myotonia has been supported by a recent randomized control trial.^100^ The most common side effect is GI distress^101^ that may improve if taken with food. Serious side effects include ventricular arrhythmias and patients should have regular EKG monitoring. Other medications, mostly affecting sodium channels, have shown varying success, including carbamazepine, phenytoin, procainamide, and flecainide.^87^ Acetazolimide may work well in acetazolamide-responsive myotonia^91^ but rarely reported to cause paralysis in PMC.^102^^,^^103^

## Conclusion

The myotonic disorders are a heterogeneous group of diseases that result in clinical and/or electrical myotonia. The resulting severity can range from asymptomatic electrical myotonia, as in some cases of dominant MC, to severe disability as in advanced DM1 or myotonia permanens. Correct diagnosis is important for genetic counseling, treatment and proper screening for systemic features. Currently, treatment remains symptomatic, but research in therapies that target the genetic or molecular pathophysiology of these diseases is ongoing.^104^^-^^10^

## Notes:

**How to cite this article:** Hahn C, Salajegheh MK. Myotonic disorders: A review article. Iran J Neurol 2016; 15(1): 46-53**.**
